# *Lactobacillus plantarum* Attenuates Cadmium-Induced Intestinal Barrier Dysfunction and Systemic Toxicity in Broiler Chickens: Association with Calcium Homeostasis, PIEZO1 Expression, and Tight-Junction Integrity

**DOI:** 10.3390/toxics14080727

**Published:** 2026-08-16

**Authors:** Farah Ijaz, Muhammad Farhan Rahim, Mohammed A. Bakr, Muhammad Ali Khan, Hafeez Ur Rehman Ali Khera, Yan Li, Chuxian Quan, Hang Su, Miao An, Shaokat Ali, El Fatihi Imad, Jiakui Li

**Affiliations:** 1 College of Animal Science and Veterinary Medicine, Huazhong Agricultural University, Wuhan 430070, China; farahijaz0786@webmail.hzau.edu.cn (F.I.); farhan092@webmail.hzau.edu.cn (M.F.R.); mohamed.bakr2040@yahoo.com (M.A.B.); muhammad_ali@webmail.hzau (M.A.K.); drhafeezkhera@gmail.com (H.U.R.A.K.); yanli.vet@webmail.hzau.edu.cn (Y.L.); qcx2000@webmail.hzau.edu.cn (C.Q.); su_hang@webmail.hzau.edu.cn (H.S.); am13191101068@163.com (M.A.); alishaokat0786@gmail.com (S.A.); imadelfatihi3@gmail.com (E.F.I.); 2 College of Animal Science, Huazhong Agricultural University, Wuhan 430070, China; 3 Hubei Jiangxia Laboratory, Wuhan 430200, China

**Keywords:** PIEZO1, *Lactobacillus plantarum*, cadmium chloride, tight junction, hepatotoxicity

## Abstract

Heavy metal pollution is a serious environmental concern worldwide. Cadmium is one of the most common and hazardous heavy metals and is known to impair intestinal barrier integrity. Therefore, this study was designed to evaluate the protective effects of *Lactobacillus plantarum* against cadmium chloride (CdCl_2_)-induced toxicity in chickens. A total of 120 one-day-old Arbor Acres broiler chickens were randomly divided into four equal groups (*n* = 30 birds/group). Following a 4-day acclimation period, the chickens were subjected to a 28-day feeding trial. The control group (CON) received a standard basal diet, the probiotic group (LB) received *L. plantarum* at 1 × 10^8^ CFU/mL via oral gavage, the co-treatment group (LC) received *L. plantarum* at 1 × 10^8^ CFU/mL together with CdCl_2_ at 80 mg/kg, and the toxin group (CD) received CdCl_2_ at 80 mg/kg. Cadmium exposure markedly increased mortality, reduced survival rates, elevated serum liver enzyme activities (*p* < 0.0001), increased cadmium accumulation in tissues (*p* < 0.05), and decreased body weight gain in chickens (*p* < 0.0001). Moreover, cadmium exposure was associated with altered tissue Ca^2+^ homeostasis, upregulation of PIEZO1 expression and impairment of the epithelial tight-junction proteins, including ZO-1, occludin, and claudin-1. In contrast, *L. plantarum* supplementation improved intestinal barrier integrity and restored intestinal morphology, including villus height and crypt depth, which were adversely affected by cadmium exposure. Collectively, *L. plantarum* supplementation attenuated cadmium-induced systemic and intestinal toxicity, as evidenced by multiple protective mechanisms, such as reduced mortality, decreased tissue cadmium accumulation (*p* < 0.05), improved biochemical parameters, and preservation of intestinal morphology and tight-junction integrity. These findings suggest that *L. plantarum* may provide a potential dietary strategy for mitigating cadmium toxicity in broiler chickens.

## 1. Introduction

Environmental contamination with heavy metals is a major global concern [1]. Among these pollutants, cadmium is particularly hazardous because of its high toxicity, persistence, and bioaccumulative nature. Cadmium is categorized as a Group I carcinogen and is recognized as a major food contaminant by the World Health Organization [2]. Widespread environmental contamination can result from agricultural and industrial activities, including the use of phosphate fertilizers, sewage sludge, wastewater, pesticides, pigments, plastic stabilizers and nickel–cadmium batteries [3,4]. Cadmium poses a serious threat to animal and human health because it can accumulate in metabolically active organs and edible tissues, thereby adversely affecting animal health, production, and food safety [5]. Chickens are particularly susceptible to cadmium toxicity, which may arise from contaminated feed ingredients, drinking water, soil, and fertilizers, and can cause extensive gastrointestinal damage [6].

The gastrointestinal tract is a key target of cadmium toxicity because it is an important site of cadmium absorption and represents the first biological barrier against ingested toxicants [7]. Cadmium can impair intestinal homeostasis by disrupting the intestinal mucosa and epithelial cells, resulting in increased intestinal permeability and susceptibility to infection, inflammation and malabsorption [8,9]. Intestinal homeostasis is maintained through interaction among epithelial, immune, stromal and microbial components [10]. Therefore, maintaining intestinal barrier integrity is essential for limiting local intestinal damage and subsequent systemic toxicity. In this context, the intestine is a biologically relevant site for both cadmium absorption and host-microbial interaction.

Microbial bioremediation has attracted considerable interest in recent years due to its cost-effective and environmentally sustainable strategy for reducing heavy metal toxicity [11]. Among biological approaches, probiotics may alleviate cadmium-induced injury by binding metal ions, reducing intestinal absorption, modulating the gut microbiota, supporting immune responses, and preserving epithelial-barrier function [12]. Some probiotic strains, particularly members of the genera *Lactobacillus* and *Bifidobacterium,* can bind heavy metals through cell-wall components, including peptidoglycan, teichoic acids, and exopolysaccharides [13,14]. In particular, the exopolysaccharides of *Lactobacillus plantarum (L. plantarum)* have demonstrated protective effects against cadmium-induced epithelial injury [15]. Administration of *L. plantarum* and related probiotics has also been associated with improved intestinal morphology and an improved villus height-to-crypt depth ratio [16,17]. Probiotics may additionally modulate gastrointestinal inflammation and mast cell response during intestinal stress [18]. Despite these reported protective properties, whether *L. plantarum* can effectively mitigate dietary cadmium-induced intestinal barrier dysfunction in broiler chickens remains unclear. In particular, the relationship of probiotics-mediated protection to calcium homeostasis, PIEZO1 expression, mast cell infiltration and regulation of ZO-1, occludin and claudin-1 has not been sufficiently characterized in cadmium-exposed poultry. This knowledge gap provided the scientific rationale for the current study.

However, the molecular links underlying cadmium-induced epithelial damage and the protective effects of probiotics remain poorly understood. PIEZO1 is a mechanosensitive Ca^2+^-permeable ion channel involved in epithelial mechano-transduction and cellular responses to mechanical stimuli [19]. Under physiological conditions, PIEZO1 contributes to the maintenance of epithelial-barrier integrity and intercellular junctions [20]. Alterations in PIEZO1-related mechano-transduction have also been linked to increased epithelial permeability and dysfunction of the intestinal barrier [21]. However, most of the available evidence comes from non-avian systems or association-based experimental models, and direct functional evidence in broiler chickens remains limited. Building on our previous observation of cadmium-induced intestinal-barrier disruption, endoplasmic-reticulum stress, and altered PIEZO1-related responses in broiler chickens [22], the present study investigated whether similar responses occur following dietary cadmium exposure and whether they are attenuated by *L. plantarum*.

The novelty of the present study lies in evaluating dietary cadmium-induced intestinal injury in relation to tissue calcium levels, PIEZO1 expression, infiltration of mast cells, and epithelial tight-junction integrity following *L. plantarum* supplementation. Previous studies suggest that PIEZO1 may participate in the regulation of intracellular calcium homeostasis, while altered calcium signaling has been implicated in epithelial junctional dysfunction under pathological conditions [23,24]. However, because PIEZO1 activity was not directly manipulated in the present study, the observed relationships are interpreted as associations.

The tight junction proteins zonula occludens-1 (ZO-1), occludin and claudin-1 are key components of the epithelial barrier and play essential roles in maintaining intestinal homeostasis [25]. Heavy metal exposure disrupts tight-junction protein expression in chickens, thereby impairing intestinal barrier function and increasing paracellular permeability [26]. Notably, *L. plantarum* strains have been reported to enhance the expression of tight-junction proteins while reducing intestinal inflammation and permeability, highlighting their therapeutic potential for alleviating heavy metal-induced intestinal and systemic toxicity [27]. In our previous study, we found that cadmium exposure through drinking water disrupted the gut-liver axis, increased intestinal barrier leakage, and induced endoplasmic reticulum stress and altered PIEZO1-related response in broiler chickens [22]. However, whether similar effects occur following dietary cadmium exposure and whether they can be attenuated by *L. plantarum* remain unclear. The present study therefore evaluated dietary cadmium toxicity and probiotic protection in relation to calcium homeostasis, PIEZO1 expression and epithelial tight-junction integrity. Based on these observations, we hypothesized that dietary CdCl_2_ exposure would disrupt calcium homeostasis in the intestine and serum, increase PIEZO1 expression, and compromise intestinal tight-junction integrity, whereas *L. plantarum* supplementation would attenuate these effects. To test this hypothesis, broiler chickens were exposed to dietary CdCl_2_, with or without *L. plantarum* supplementation, and systemic toxicity, intestinal morphology, mast-cell abundance, calcium concentrations, PIEZO1 expression, and tight-junction proteins were evaluated.

## 2. Materials and Methods

### 2.1. Bacterial Strain and Inoculum Preparation

The *Lactobacillus plantarum* strain used in this study was isolated from Tibetan chickens and had been previously characterized and maintained in our laboratory [28]. The strain was identified by 16S rRNA gene sequencing using universal primers 27F and 1492R, which revealed 99% sequence similarity to *L. plantarum.* The corresponding BioProject accession number is PRJNA954678. For the present experiment, the strain was cultured in De Man, Rogosa, and Sharpe (MRS) broth (Qingdao Hi-Tech Industrial Park Hope Bio-Technology Co., Ltd., Qingdao, Shandong, China). The culture *L. plantarum* was serially diluted tenfold (10^−1^ to 10^−10^), and 1 mL of each dilution was plated onto MRS agar plates. The plates were incubated at 37 °C for 48 h. Based on viable bacterial counts, the bacterial inoculum administered to the chickens by oral gavage was adjusted to a final concentration of 1 × 10^8^ CFU/mL (per bird/per day). Viable bacterial counts were confirmed before administration, and fresh *L. plantarum* cultures were prepared daily from overnight MRS broth cultures.

### 2.2. Experimental Design

A total of 120 one-day-old Arbor Acres broiler chicks, with an average body weight of 43 ± 3 g, were purchased from Zhengda Poultry Co. Ltd., Xiangyang, China. The chicks were reared under standard brooding conditions at 33 ± 1 °C and 60–70% relative humidity, with a photoperiod of 23 h light and 1 h darkness. Feed and water were provided ad libitum.

After a 4-day acclimatization period, the chicks were randomly allocated to four experimental groups, with 30 birds per group. The experimental design was as follows: CON (control group), birds fed the basal diet only; LB (probiotic only group), birds fed the basal diet and given *L. plantarum* at 1 × 10^8^ CFU/mL through oral gavage; LC (Probiotic+ CdCl_2_ group), birds fed the basal diet supplemented with CdCl_2_ at 80 mg/kg and given *L. plantarum* at 1 × 10^8^ CFU/mL through oral gavage; and CD (CdCl_2_ only group), birds fed the basal diet supplemented with CdCl_2_ at 80 mg/kg.

Cadmium chloride (CdCl_2_; analytical reagent grade, 99% purity; molecular weight, 183.32) was obtained from Shanghai Macklin Biochemical Co., Ltd., Shanghai, China. A dietary CdCl_2_ concentration of 80 mg/kg was selected to establish a reproducible short-term cadmium-toxicity model. Previous studies have reported on cadmium-associated toxicity in poultry following dietary exposure [29]. In particular, a previous broiler study reported significant hematobiochemical and histopathological alterations following exposure to an analogous cadmium dose of 75 mg/kg/day [30]. Therefore, 80 mg/kg CdCl_2_ was selected for the present 28-day dietary exposure model. However, the present experimental design did not exactly reproduce the previously reported protocol. A dose-ranging pilot study was not conducted, and this limitation is acknowledged in the discussion.

The concentration of *L. plantarum* selected was 1 × 10^8^ CFU/mL based on the study of Wang et al. [28]. They reported measurable effects on broiler growth performance and gut microbiota. A dose-ranging pilot study was not conducted, as stated in the study limitations.

The basal feed was obtained from Zhengda Feed Company (Xiangyang, China). The background cadmium concentration in the basal diet was 0.38 mg/kg, as determined by graphite furnace atomic absorption spectrometry using an Agilent 240Z GFAAS system Agilent Technologies, Inc., Santa Clara, California, USA. This concentration was below the maximum limit of 0.5 mg/kg specified in the Chinese National Feed Hygiene Standard (GB 13078-2017, Hygienical Standard for Feeds. Standards Press of China: Beijing, China, 2017) and was therefore considered a low background concentration rather than an additional experimental source of cadmium exposure.

### 2.3. Sample Collection and Growth Performance

During the 28-day experimental period, body weight was recorded weekly using a digital precision scale. At each sampling time point (day 14 and day 28), half of the birds from each group were randomly selected for blood and tissue collection. Blood samples were collected from the brachial vein using a 22-gauge needle and allowed to clot at room temperature. Samples were then centrifuged at 3000× *g* for 10 min at 4 °C using an HC-2518 centrifuge, and the separated serum was stored at −20 °C until further analysis.

Following blood collection, the chickens were euthanized by cervical dislocation, and tissue samples, including liver, cecum, kidney, and tibia, were collected. Liver, cecum and kidney samples were randomly divided into two portions: one portion was fixed in 4% paraformaldehyde (G1101, Servicebio, Wuhan, China) for histopathological analysis, while the other portion was immediately snap-frozen in liquid nitrogen and stored at −80 °C for molecular analyses. Tibial morphometric parameters were assessed using a digital caliper, while bone density and volume were measured using a bone densitometer InAlyzer, OsteoSys Co., Ltd., Seoul, Republic of Korea. For molecular and biochemical analyses, at least three to four biological replicates per group were used, and technical measurements were performed in triplicate. The study protocol was reviewed and approved by the Animal Welfare and Ethics Committee of Huazhong Agricultural University (HZAUCH-2025-0055). All experimental procedures were conducted in accordance with the ARRIVE guidelines.

### 2.4. Determination of Cadmium Concentrations in Intestine

Cadmium concentrations in cecal tissues were determined using graphite furnace atomic absorption spectrometry (GFAAS; Agilent 240Z AA, Agilent Technologies, Inc., Santa Clara, CA, USA) following a modified US EPA Method 3050B. Briefly, approximately 0.3 g of each cecal tissue sample was weighed and transferred into polytetrafluoroethylene (PTFE) digestion tubes containing 5 mL of 68% ultrapure nitric acid (HNO_3_) for overnight predigestion at room temperature. Samples were then heated at 120 °C until complete digestion. After cooling to room temperature, the digests were diluted to a final volume of 25 mL with ultrapure deionized water. Cadmium concentrations were quantified using certified cadmium standard solutions for calibration and expressed as μg/g tissue.

### 2.5. Calcium Assay

Calcium concentrations in tissue homogenates were determined using a commercial analytical kit (HYCEZMBIO, Wuhan, China) based on the Arsenazo III colorimetric method. Calcium ions reacted with Arsenazo III to form a purple-blue complex, and the absorbance was measured at 650 nm using an automated biochemical analyzer (BS-240 Vet, Shenzhen Mindray Animal Medical Technology Co., Ltd., Shenzhen, China). Calcium concentrations were calculated from a standard calibration curve. Total protein concentrations in the same tissue homogenates were determined using the bicinchoninic acid (BCA) assay, and calcium concentrations were normalized to protein content. Results were expressed as mmol Ca^2+^/g protein.

### 2.6. Biochemical Analysis

Serum biochemical parameters were measured using commercial assay kits according to the manufacturers’ instructions. Measurements were performed using a BS-240VET automatic biochemical analyzer (Mindray Animal Medical Technology Co., Ltd., Shenzhen, China). The analyzed parameters included liver function markers, namely alkaline phosphatase (ALP), alanine aminotransferase (ALT), and aspartate aminotransferase (AST), as well as renal and metabolic indicators, including total protein (TP) and creatinine (CREA).

### 2.7. Hematoxylin and Eosin (H & E) Staining

Tissue samples fixed in 4% paraformaldehyde were dehydrated through a graded ethanol series, cleared in xylene (Sinopharm Chemical Reagent Co., Ltd., Shanghai, China), and embedded in paraffin wax. Paraffin-embedded tissues were sectioned at a thickness of 4–5 μm and stained with hematoxylin and eosin (H&E) according to standard histological procedures, as previously described. The stained sections were examined for histopathological changes using an inverted microscope (Olympus IX3-H035D, Olympus Corporation, Tokyo, Japan). Histological images were quantified using ImageJ software (ImageJ, version 1.54p, National Institutes of Health, Bethesda, MD, USA) by an observer blinded to treatment allocation.

### 2.8. Quantitative Real-Time PCR (RT-qPCR)

Total RNA was isolated from approximately 100 mg of tissues using TRIzol reagent (Life Technologies Corporation, Carlsbad, CA, USA). RNA concentration and purity were assessed by measuring the absorbance ratio at 260/280 nm with a Nanodrop 2000 spectrophotometer (Thermo Fisher Scientific Inc., Waltham, MA, USA). One microgram of total RNA was reverse transcribed using the HiScript II Q RT SuperMix with gDNA Wiper kit (Vazyme Biotech Co., Ltd., Nanjing, China) according to the manufacturer’s instructions. Quantitative reverse-transcription PCR (RT-qPCR) was performed using Taq Pro Universal SYBR Green Master Mix (Vazyme Biotech Co., Ltd., Nanjing, China) on a Step OnePlus™ Real-Time PCR System (Applied Biosystems, Foster City, CA, USA). The amplification protocol consisted of an initial denaturation of 30 s at 95 °C followed by 40 cycles of denaturation of 10 s at 95 °C, annealing of 30 s at 60 °C, and extension of 30 s at 60 °C. Amplification specificity was assessed by melting curve analysis at 95 °C for 15 s, 60 °C for 60 s and 95 °C for 15 s. GAPDH was used as the housekeeping gene, and relative gene expression was calculated using the 2^−ΔΔCq^ method. Primers sequences were designed based on sequences obtained from the National Center for Biotechnology Information (NCBI) database and are listed in Table 1.

### 2.9. Western Blot Analysis

Tissue samples stored at −80 °C were homogenized in ice-cold RIPA lysis buffer (Servicebio, Wuhan, China) supplemented with protease and phosphatase inhibitors. The homogenates were centrifuged at 12,000× *g* for 15 min at 4 °C, and the supernatants were collected for protein analysis. Protein concentration was determined using a bicinchoninic acid (BCA) protein assay kit (P00112-1, Beyotime Biotechnology, Shanghai, China). Equal amounts of protein were mixed with loading buffer, denatured and separated by SDS-PAGE using polyacrylamide gels of different concentrations (6%, 8%, 10% and 12%), depending on the molecular weight of the target protein. A total of 20 µg of protein was loaded per lane. A pre-stained protein marker (ABclonal, RM19001, Wuhan, China) was included to monitor protein separation.

Following electrophoresis, proteins were transferred onto polyvinylidene difluoride (PVDF) membranes (Merck Millipore, Burlington, MA, USA). To prevent nonspecific binding, the membranes were blocked with 5% skim milk prepared in TBST (Tris-buffered saline with 0.1% Tween 20) for 90 min at room temperature and then incubated overnight (14–16 h) at 4 °C with the appropriate primary antibodies. After three washes with TBST for 10 min each, membranes were incubated with a horseradish peroxidase (HRP)-conjugated secondary antibody (goat anti-rabbit IgG, H + L, AS014, 1:8000; ABclonal, Wuhan, China) for 60–90 min at room temperature. Membranes were subsequently washed three times with TBST for 10 min each. Protein bands were visualized using enhanced chemiluminescence (ECL) substrate (MA0186-1, MeilunBio, Dalian, China) and an imaging system (FUSION Solo S, Vilber, Collégien, France) Band intensities were quantified using the Image J software (National Institutes of Health (NIH), Bethesda, MD, USA), with β-actin used as the loading control. Detailed information on the primary antibodies is provided in Table 2.

### 2.10. Immunohistochemistry Staining

Tissues were fixed in 4% paraformaldehyde, embedded in paraffin, sectioned at a thickness of 4 μm using a rotary microtome, and mounted on adhesive glass slides. Sections were deparaffinized twice in xylene for 10 min each and rehydrated through a descending ethanol series (100%, 95%, 80%, and 70%) for 5 min at each concentration, followed by rinsing in distilled water. Antigen retrieval was performed in 0.01 M citrate buffer (pH 6.0) for 20 min at 95 °C. After cooling at room temperature, endogenous peroxidase activity was quenched with 3% hydrogen peroxide for 10 min. Sections were then incubated with blocking buffer for 1 h at room temperature to reduce nonspecific antibody binding.

Sections were subsequently incubated overnight at 4 °C with the following primary antibodies diluted in PBS containing 1% bovine serum albumin (BSA): anti-occludin (1:200, WL01996), anti-ZO-1 (1:200, WL03419), anti-claudin-1 (1:200, WL03073), and anti-FAM38A/PIEZO1 (1:500, A23380). After washing with PBS, the sections were incubated with HRP-conjugated secondary antibodies (GB23204) for 50 min at room temperature. The immunoreactive signals were visualized using 3,3-diaminobenzidine (DAB) substrate, followed by hematoxylin counterstaining. Sections were then differentiated, blued, dehydrated, cleared, and mounted with neutral balsam. Negative controls were processed in parallel under identical conditions, except that the primary antibody was omitted. Images were acquired using a light microscope (Olympus IX3-H035D, Olympus Corporation, Tokyo, Japan), and immunohistochemical staining was analyzed quantitatively by ImageJ software (version 1.54p, National Institutes of Health, Bethesda, MD, USA).

### 2.11. Immunofluorescent Analysis

After deparaffinization and rehydration, paraffin-embedded tissue sections were subjected to antigen retrieval using an EDTA-based buffer. Sections were then permeabilized with 0.3% Triton X-100 to facilitate antibody penetration and blocked with 5% BSA for 25 min at room temperature to reduce nonspecific binding. Sections were subsequently incubated with the appropriate primary antibodies, followed by fluorophore-conjugated secondary antibodies. Nuclei were counterstained with DAPI for 5–10 min in the dark at room temperature. Fluorescence signals were visualized using a fluorescence microscope (EVOS FL, Thermo Fisher Scientific, USA).

### 2.12. Toluidine Blue Staining

Mast cells in tissues were detected using toluidine blue staining. Briefly, tissue sections were deparaffinized twice in xylene for 10 min each and rehydrated through a graded ethanol series. The sections were then immersed in toluidine blue staining solution (pH 4.0) for 5 min and rinsed briefly with distilled water, differentiated in 95% ethanol for 1 min, dehydrated twice in 100% ethanol for 3 min each, cleared in xylene twice for 5 min each, and mounted with coverslips using neutral balsam. Mast cells were identified by their metachromatic granules and counted using a light microscope at ×400 magnification. To minimize the effect of regional differences in mast cell distribution, five non-overlapping high-power fields (HPFs) were randomly selected from each intestinal section. The number of mast cells in each field was counted and the mean number of mast cells per HPF per bird was used for statistical analysis. To minimize potential observer bias, cell quantification was performed by an observer blinded to the experimental groups.

### 2.13. Statistical Analysis

All data from each sampling time point (day 14 and day 28) were analyzed separately using two-way analysis of variance (ANOVA), with cadmium exposure and probiotic supplementation as the two independent factors, followed by Tukey’s multiple comparisons test in GraphPad Prism version 8.0.2 (GraphPad Software, Inc., San Diego, CA, USA). Data normality was assessed using the Shapiro–Wilk test prior to ANOVA. Survival and mortality data were analyzed using the Kaplan–Meier method. Quantitative data were presented as mean ± standard deviation (SD). For molecular and protein expression analysis, a minimum of 3–4 biological replicates were used, and assays were performed in technical triplicates. A two-tailed *p* value < 0.05 was considered statistically significant. Pearson’s correlation coefficient (r) was used to evaluate the strength and direction of associations between variables, with statistical significance determined by the corresponding *p* value. All histological image quantifications were performed by an observer blinded to the experimental group allocation.

## 3. Results

### 3.1. Effects of Cadmium and L. plantarum on Growth Performance, Organ Indices, Tissue Cadmium Accumulation, Survival, and Calcium Homeostasis

**Figure 1:**
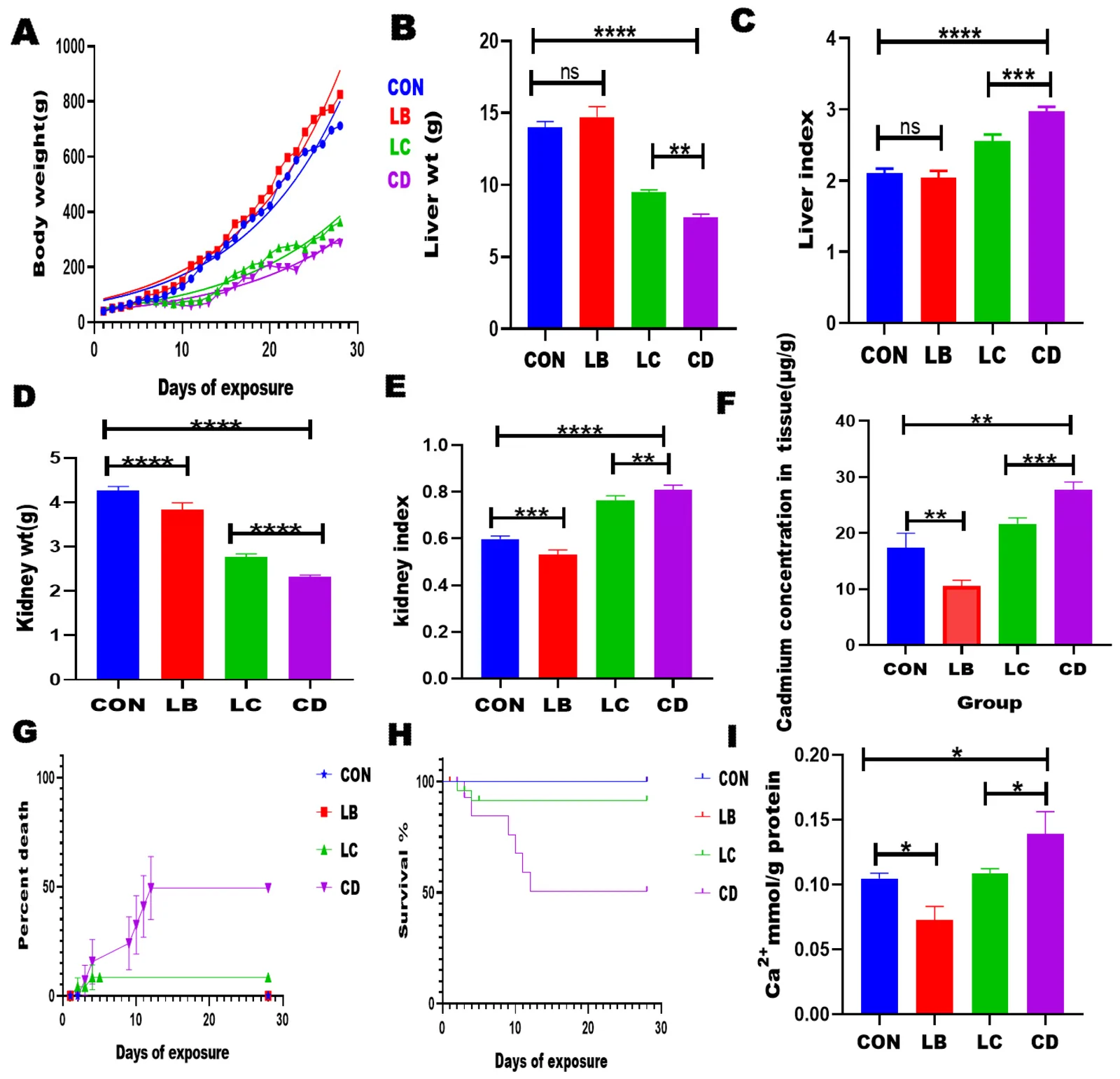
Effects of *L. plantarum* and Cadmium on growth, organ indices, mortality, survival, and cecal cadmium and Ca^2+^ concentration following CdCl_2_. (**A**) Body-Weight; (**B**) Liver weight percent; (**C**) Liver index; (**D**) kidney weight; (**E**) kidney index; (**F**) Cadmium concentration in cecal tissues; (**G**) Mortality rate; (**H**) Survival probability; (**I**) Cecal Ca^2+^ concentration. Asterisks denote significant differences. Significance level stated the values among individual groups as means ± SD, * *p* < 0.05, ** *p* < 0.01, *** *p* < 0.001, **** *p* < 0.0001, ns: non-significant.

The body weight, organ indices, tissue concentration, survival, mortality and cecal calcium concentrations were compared among the CON, LB, LC and CD groups. All groups exhibited a steady gain in body weight over the 28-day period; however, cadmium exposure significantly impaired growth, with the CD group showing the lowest body weight compared with the CON group (*p* < 0.0001). Conversely, the LC group showed significantly greater body weight relative to the CD group, although body weight remained lower than that of the CON and LB groups. Importantly, broilers fed *L. plantarum* exhibited greater body weight than the CON group, suggesting a potential growth-promoting effect of *L. plantarum* supplementation (Figure 1A). Cadmium exposure also significantly altered liver and kidney parameters. Absolute liver weight was significantly lower in the LC and CD groups than in the CON and LB groups, with no significant difference being observed between the CON and LB groups (Figure 1B). However, the liver index was significantly elevated in the CD group compared with the CON group (*p* < 0.0001), indicating liver morphology associated with cadmium exposure. This elevation was significantly attenuated in the LC group, indicating that cadmium-induced liver damage was mitigated by supplementation with *L. plantarum*, whereas the LB group did not differ significantly from the CON group (Figure 1C). Similarly, cadmium exposure significantly decreased absolute kidney weight and increased the kidney index (*p* < 0.0001), indicating renal injury and pathological alterations. These changes were partially improved by *L. plantarum* supplementation in the LC group but did not completely return to control levels (Figure 1D,E). Tissue cadmium concentrations were highest in the CD group and were significantly lower in the LC group (*p* < 0.001). This finding suggests that *L. plantarum* supplementation may reduce cadmium accumulation in tissues, potentially through decreased intestinal cadmium absorption and/or enhanced elimination (Figure 1F). The survival curves and Kaplan–Meier analysis consistently showed that cadmium exposure increased mortality and decreased survival probability. The CON and LB groups had 100% survival throughout the experimental period. Importantly, *L. plantarum* supplementation improved the survival of cadmium-exposed birds, as the LC group exhibited lower mortality and a higher survival probability than the CD group (Figure 1G,H). Exposure to cadmium also increased cecal Ca^2+^ levels, suggesting disruption of intestinal calcium homeostasis. This increase was associated with altered cecal calcium homeostasis and increased PIEZO1 expression during cadmium-induced intestinal injury. *L. plantarum* supplementation significantly lowered the cecal Ca^2+^ concentrations in the LC group compared with the CD group (Figure 1I), indicating partial restoration of cecal homeostasis. Collectively, these results indicate that *L. plantarum* supplementation improved growth performance, reduced mortality, decreased tissue cadmium accumulation, and partially restored cecal calcium homeostasis during cadmium exposure.

### 3.2. Probiotic Supplementation Mitigates Cadmium-Induced Hepatorenal Toxicity and Preserves Systemic Metabolic Balance

**Figure 2:**
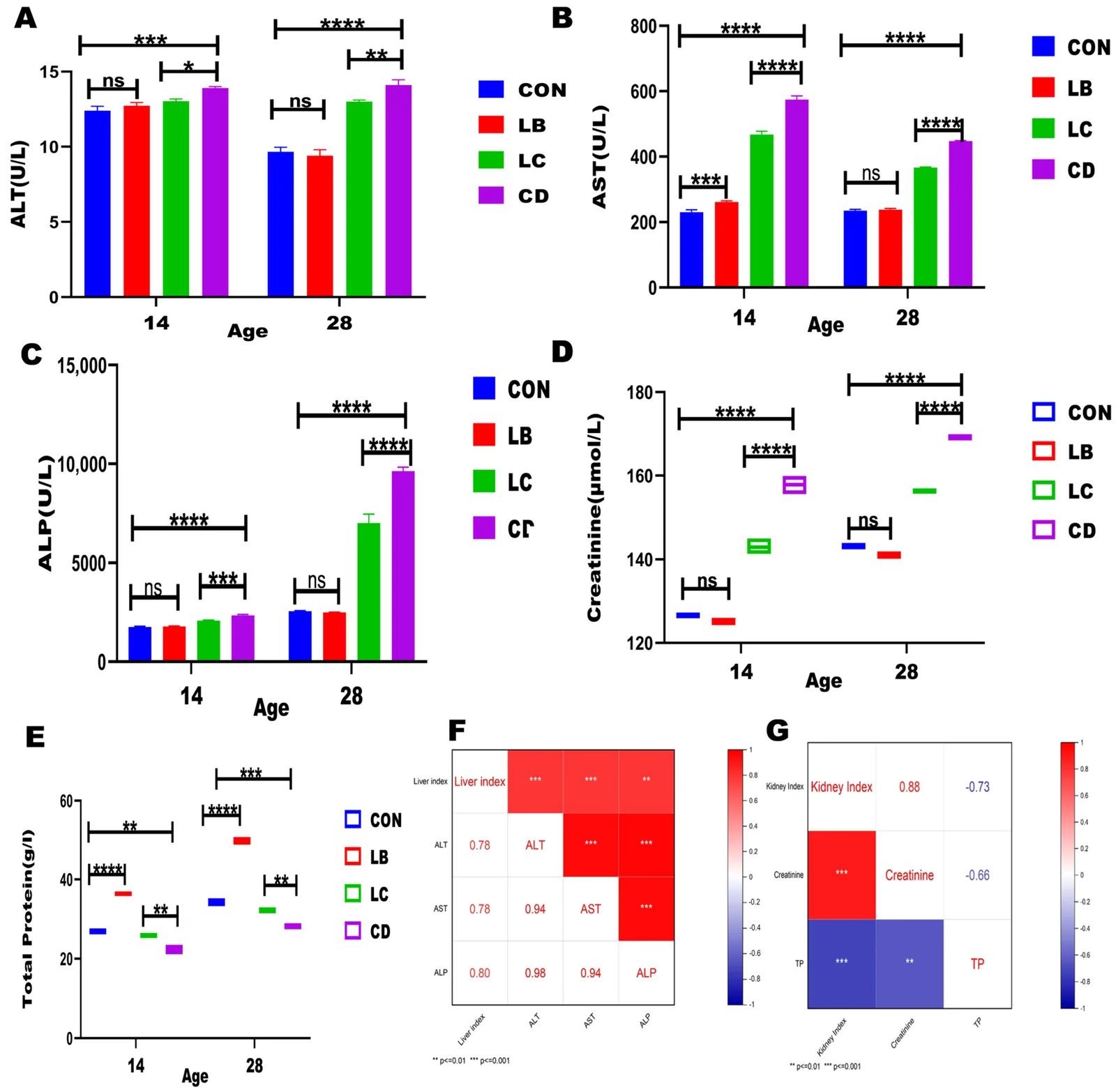
Effects of CdCl_2_ exposure and *L. plantarum* supplementation on serum biochemical parameters and organ- functions association. (**A**) Alanine aminotransferase (ALT) (**B**) Aspartate aminotransferase (AST) (**C**) Alkaline phosphatase (ALP) (**D**) Serum creatinine (**E**) Total protein (**F**) Pearson correlation analysis between Serum liver enzymes and liver index (**G**) Pearson correlation among kidney index, creatinine and total protein. Asterisks indicate statistically significant differences among groups. Data are presented as means ± SD, * *p* < 0.05, ** *p* < 0.01, *** *p* < 0.001, **** *p* < 0.0001, ns: non-significant.

Systemic protective effects of *L. plantarum* were further assessed by the serum biochemical markers of liver and renal function, including ALT, AST, ALP, creatinine and total protein concentrations, on days 14 and 28 (Figure 2). Cadmium exposure markedly disrupted hepatic function, as reflected by increased serum ALT, AST, and ALP activities, with the most pronounced elevations observed in the CD group compared with the CON group on day 28 (*p* < 0.0001) for nearly all measured enzymes. These changes are consistent with hepatocellular and hepatobiliary injury following cadmium challenge. In contrast, birds receiving *L. plantarum* supplementation during cadmium exposure showed significantly lower ALT, AST, and ALP activities than the CD group, although enzyme levels remained above those of the CON and LB groups. Notably, *L. plantarum* alone did not increase hepatic enzyme activities, indicating that *L. plantarum* alone did not adversely affect the measured serum liver enzymes under the conditions of this study (Figure 2A–C). Cadmium exposure also impaired renal function, as indicated by significantly increased serum creatinine at both time points. The CD group showed the highest creatinine levels compared with the CON group (*p* < 0.0001), especially on day 28. The LC group showed lower creatinine concentrations than the CD group, suggesting attenuation of cadmium-associated renal dysfunction by *L. plantarum* supplementation (Figure 2D). In parallel, serum total protein was markedly reduced in cadmium-exposed birds, suggesting altered protein metabolism and impaired hepatic synthetic function following cadmium exposure. *L. plantarum* supplementation significantly improved total protein concentrations in the LC group compared with the CD group (*p* < 0.01), while the LB group showed the highest total protein levels, further supporting the potential beneficial function of *L. plantarum* in maintaining protein metabolic balance (Figure 2E). Pearson correlation analysis showed that the liver enzymes were strongly positively correlated with ALT (*p* < 0.001), AST (*p* < 0.001), and ALP (*p* < 0.01), while the three liver enzymes were also positively correlated with one another (*p* < 0.001) (Figure 2F). Similarly, kidney index and creatinine were positively correlated with each other (*p* < 0.001), whereas both were negatively associated with total protein (*p* < 0.001 and *p* < 0.01, respectively) (Figure 2G). These correlations should be interpreted as descriptive associations because variables were concurrently influenced by the experimental treatments, particularly cadmium exposure. Collectively, these results demonstrate that cadmium exposure was associated with pronounced hepatic and renal injury, whereas *L. plantarum* supplementation attenuated these biochemical alterations and preserved systemic metabolic homeostasis, preserved metabolic stability, and improved host physiological resilience against cadmium toxicity.

### 3.3. L. plantarum Preserves Cecal Architecture and Limits Mast-Cell Associated Inflammation During Cadmium Exposure

**Figure 3:**
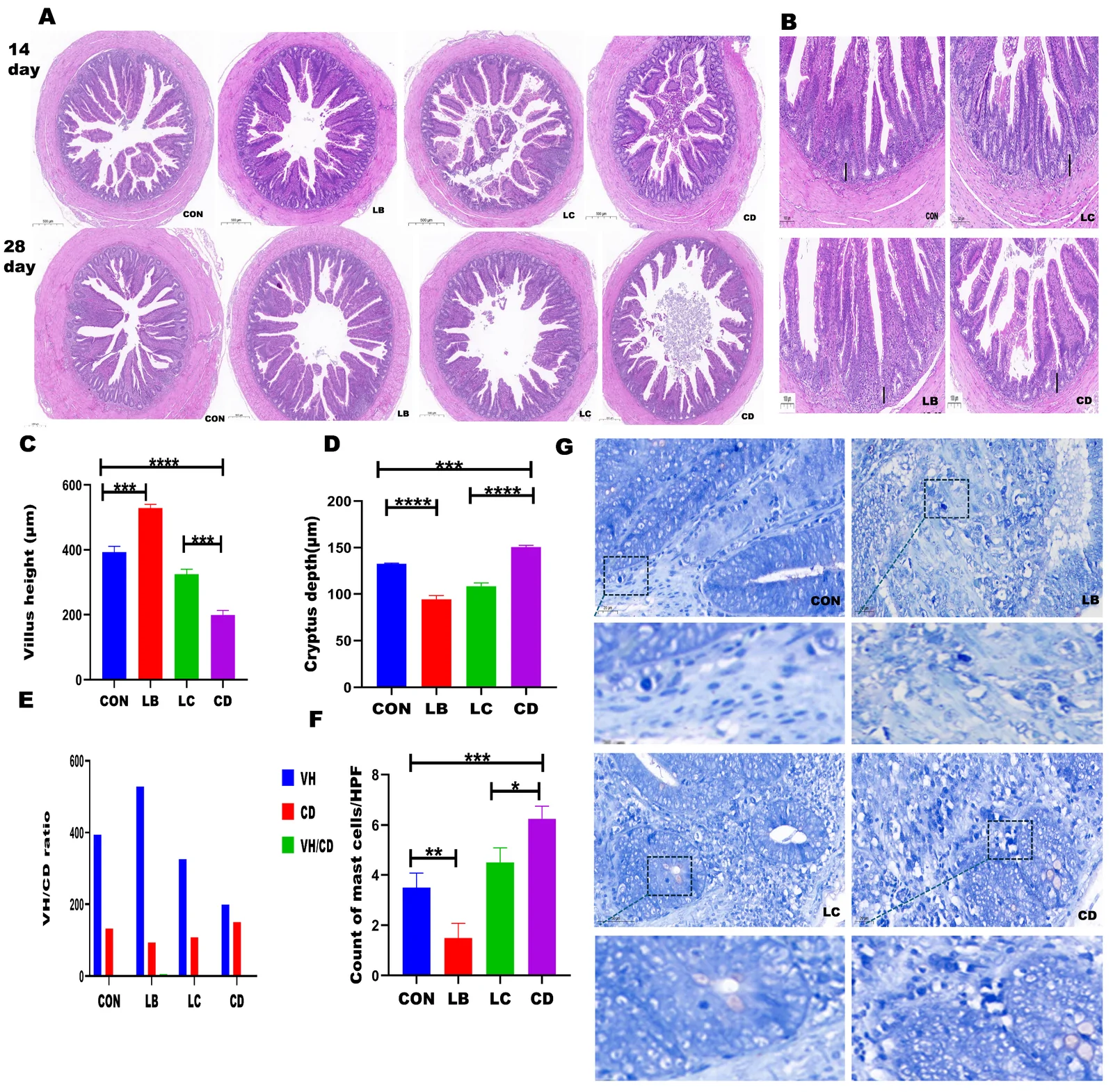
*L. plantarum* supplementation attenuates cadmium-induced cecal morphological alteration and mast cell accumulation (**A**) Representative H&E-stained cecal sections from each experimental group at days 14 and 28; scale bar = 500 µm (**B**) Representative images showing cecal crypt morphology; scale bar = 100 µm (**C**) Quantification of villus height (**D**) Quantification of crypt depth. (**E**) Villus height-to-crypt depth VH/CD ratio (**F**) Quantification of mast cell number (**G**) Representative images of mast cell in cecum; scale bar = 20 µm. Asterisks indicate Significance level stated the values among individual groups as means ± SD * *p* < 0.05, ** *p* < 0.01, *** *p* < 0.001, **** *p* < 0.0001, ns: non-significant.

Histological analysis of the cecum showed that cadmium exposure caused substantial epithelial and mucosal alterations, whereas *L. plantarum* supplementation significantly preserved intestinal morphology. H&E staining showed well-preserved mucosal folds, epithelial lining, and crypts in the CON and LB groups at both day 14 and day 28. In contrast, the CD group exhibited marked structural disruption at both time points, characterized by shortened villi, enlarged luminal spaces, epithelial damage, distorted crypts, and loss of mucosal integrity (Figure 3A,B). These observations were supported by quantitative morphometric analysis. Cadmium exposure significantly decreased villus height and increased crypt depth, suggesting decreased absorptive capacity and compensatory epithelial remodeling. Villus height and crypt depth in the LC group differed significantly from the CD group (*p* < 0.001 and *p* < 0.0001, respectively), indicating partial restoration of mucosal architecture following *L. plantarum* supplementation (Figure 3C,D). Consistent with these findings, the villus height-to-crypt depth ratio was significantly decreased following cadmium exposure and significantly increased following *L. plantarum* supplementation, indicating healing of the functional mucosal architecture (Figure 3E). Notably, *L. plantarum* alone was associated with greater villus height and a favorable mucosal profile compared with the CON group. Cadmium exposure was also associated with increased mast cell infiltration in the cecal mucosa, with the highest number of mast cells observed in the CD group. Mast cell numbers were significantly lower in the LC group compared with the CD group (*p* < 0.05), whereas the LB group showed the lowest mast cell counts (Figure 3F,G). All these findings indicate that cadmium exposure disrupted cecal morphology and increased mast cell accumulation, while *L. plantarum* supplementation was associated with preservation of mucosal architecture and attenuation of mast cell accumulation.

### 3.4. L. plantarum Attenuates Cadmium-Associated PIEZO1 Upregulation in the Cecal Epithelium

**Figure 4:**
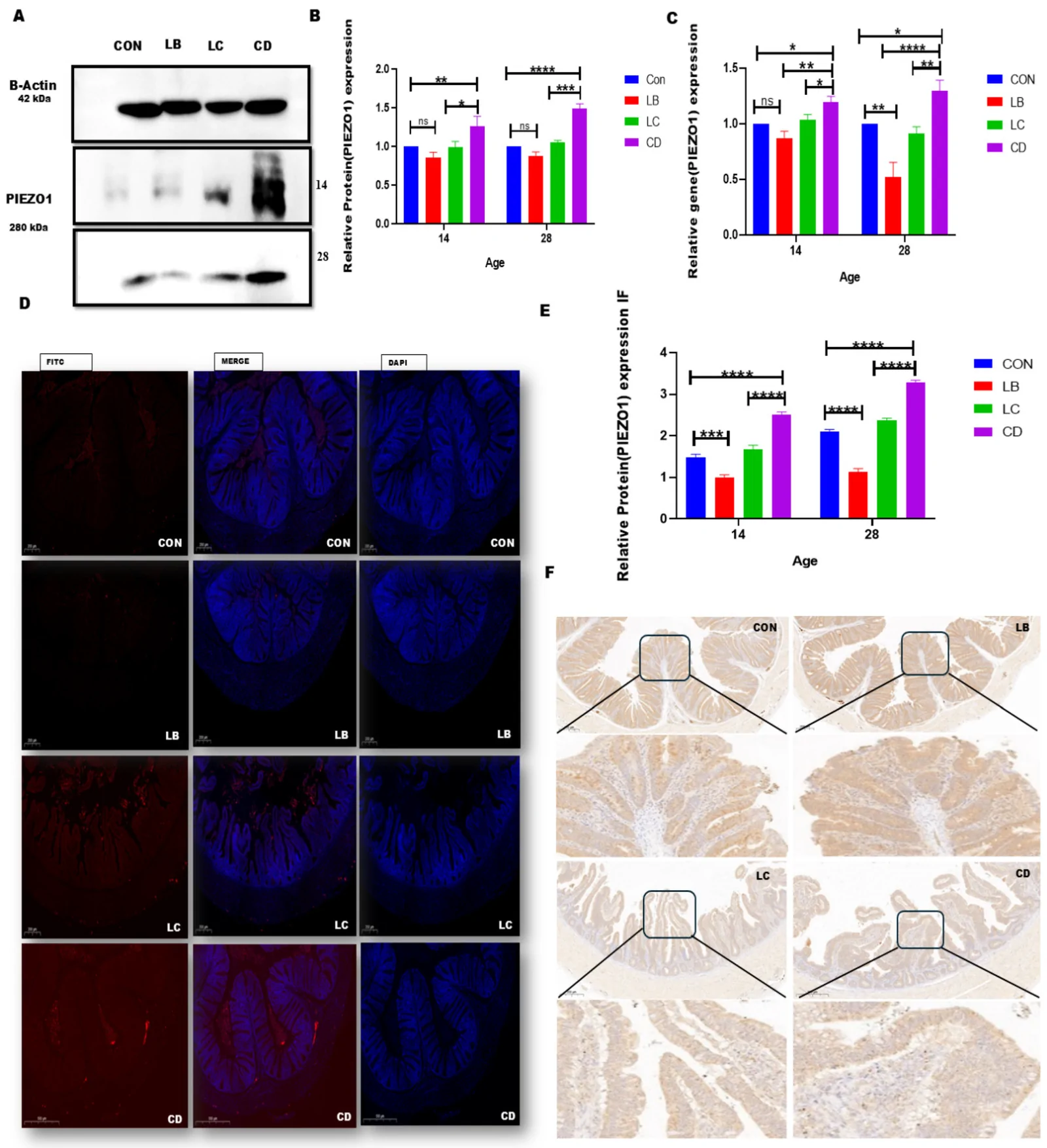
Impact of *L. plantarum* on PIEZO1 expression and localization in cecal tissue following CdCl_2_ exposure. (**A**) Western blot analysis of PIEZO1 expression with β-actin as the loading control (**B**) Densitometric quantification of PIEZO1 protein expression (**C**) Relative PIEZO1 mRNA expression by RT-qPCR (**D**) Representative immunofluorescence images of PIEZO1 in the cecal sections with DAPI; scale bar = 500 μm (**E**) Quantification of PIEZO1 immunofluorescence intensity (**F**) Representative immunohistochemical staining for PIEZO1; scale bar = 200 μm. Asterisks denote significant differences. Significance level stated the values among individual groups are shown as means ± SD, * *p* < 0.05, ** *p* < 0.01, *** *p* < 0.001, **** *p* < 0.0001, ns: non-significant.

PIEZO1, a Ca^2+^-permeable mechanosensitive ion channel, was evaluated to determine whether cadmium-induced intestinal injury was associated with altered PIEZO1 expression. Western blot analysis showed that cadmium exposure markedly increased PIEZO1 protein abundance in cecal tissue, with the strongest increase observed in the CD group, particularly on day 28. In contrast, *L. plantarum* supplementation significantly reduced PIEZO1 protein abundance in cadmium-exposed birds compared with the CD group (*p* < 0.001), indicating attenuation of cadmium-associated PIEZO1 upregulation. The LB group exhibited the lowest PIEZO1 expression, suggesting that *L. plantarum* alone showed a partial reduction in PIEZO1 expression (Figure 4A,B). Consistent with the protein findings, PIEZO1 mRNA expression was significantly increased in the CD group compared with the CON group on both day 14 and day 28, whereas the LC group showed significantly lower expression than the CD group (*p* < 0.01). PIEZO1 mRNA expression was also significantly lower in the LB group than in the CD group (*p* < 0.0001), particularly on day 28 (Figure 4C). Immunofluorescence staining further supported these findings, showing stronger PIEZO1 fluorescence in the cecal epithelium of cadmium-exposed birds, while tissues from the CON and LB groups displayed relatively weak staining. The LC group exhibited intermediate fluorescence intensity, supporting partial attenuation of cadmium-induced PIEZO1 upregulation (Figure 4D,E). Immunohistochemical analysis demonstrated a similar expression pattern, with stronger PIEZO1 immunoreactivity in the CD group and reduced staining in the LC group, whereas CON and LB tissues showed relatively limited epithelial staining (Figure 4F). Concurrently, these results demonstrate that cadmium exposure was associated with increased PIEZO1 expression in the cecal mucosa. While *L. plantarum* supplementation attenuated this response, suggesting that its protective effect against cadmium-induced injury may involve normalization of PIEZO1/Ca^2+^ level and preservation of epithelial barrier integrity.

### 3.5. L. plantarum Preserves ZO-1 Associated Tight-Junction Integrity During Cadmium-Induced Intestinal Injury

**Figure 5:**
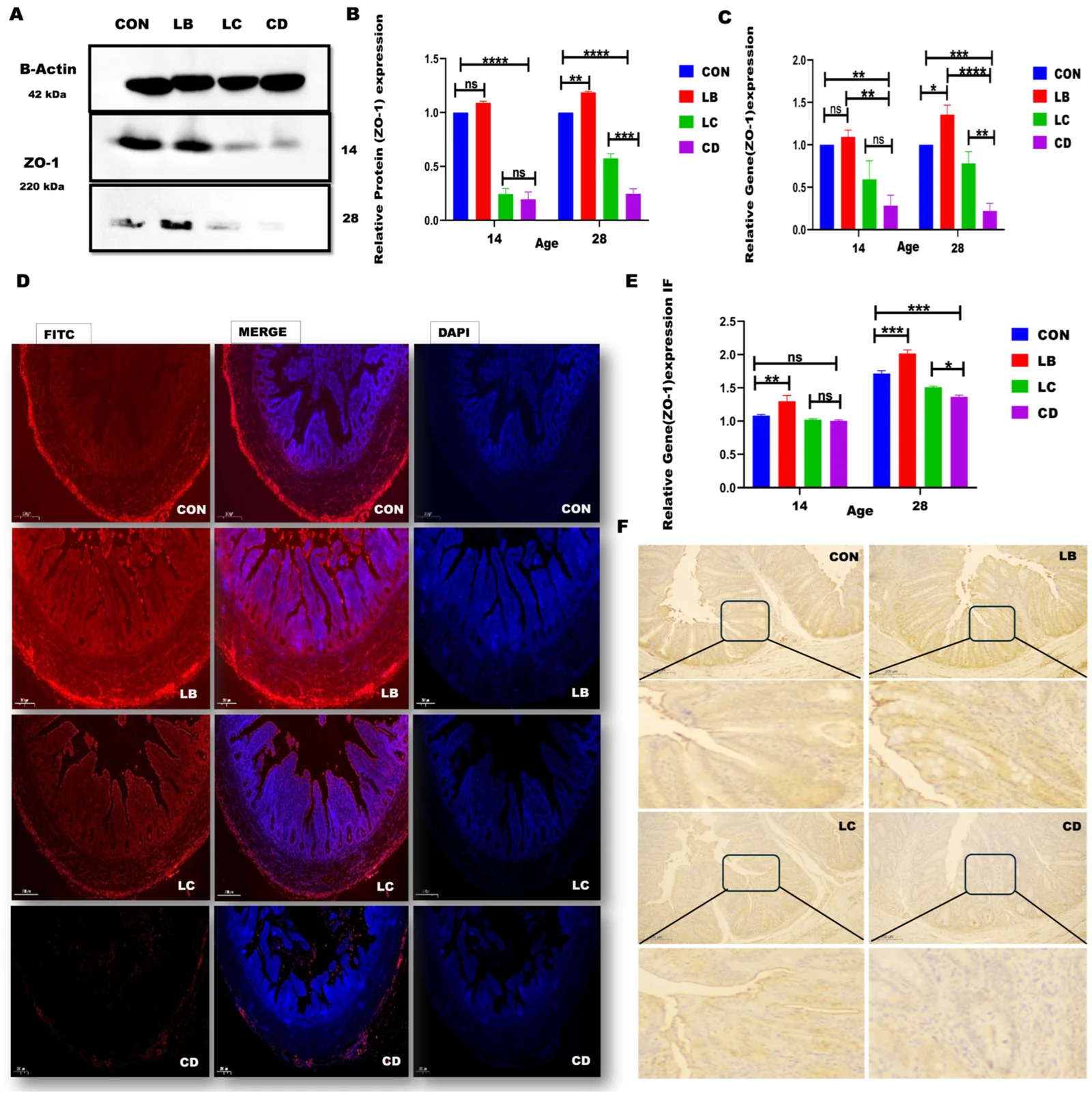
Effects of CdCl_2_ exposure and *L. plantarum* supplementation on ZO-1 expression in cecal tissue. (**A**) Western blot analysis of ZO-1 with β-actin as the loading control (**B**) Densitometric quantification of ZO-1 protein expression (**C**) Relative ZO-1 mRNA expression determined by RT-qPCR (**D**) Representative immunofluorescence images for ZO-1 in cecal sections with DAPI counterstaining; scale bar = 500 μm (**E**) Quantification of ZO-1 IF intensity (**F**) Representative immunohistochemical staining for ZO-1; scale bar = 200 μm. Significance level stated the values among individual groups are shown as means ± SD * *p* < 0.05, ** *p* < 0.01, *** *p* < 0.001, **** *p* < 0.0001, ns: non-significant.

ZO-1 was evaluated as a marker of intestinal tight-junction integrity following cadmium exposure. Western blot analysis showed strong ZO-1 protein expression in the CON and LB groups, with the highest expression observed in the LB group, particularly on day 28. In contrast, cadmium exposure markedly reduced ZO-1 protein abundance, with the lowest levels observed in the CD group. Notably, *L. plantarum* supplementation significantly increased ZO-1 protein expression in cadmium-exposed birds (LC) compared with the CD group (*p* < 0.001), particularly on day 28 (*p* < 0.001), particularly on day 28 (Figure 5A,B). Consistent with the protein data, ZO-1 mRNA expression was maintained or increased in the LB group but was significantly reduced in the CD group when compared with the LB group (*p* < 0.0001), especially on day 28. The LC group showed higher ZO-1 mRNA expression than the CD group (*p* < 0.01), indicating that *L. plantarum* preserved cadmium-induced tight-junction downregulation of ZO-1 expression (Figure 5C). Immunofluorescence staining further supported these findings, showing continuous and relatively intense ZO-1 staining along the cecal mucosa in the CON and LB groups, whereas the CD group exhibited weaker and more discontinuous staining. The LC group retained a stronger ZO-1 fluorescence signal than the CD group, consistent with partial preservation of junctional organization (Figure 5D,E). Immunohistochemical staining showed a similar pattern, with preserved ZO-1 immunoreactivity in the CON and LB groups and marked reduced staining following cadmium exposure. ZO-1 immunoreactivity was higher in the LC group than in the CD group (Figure 5F). Together, these findings demonstrate that cadmium exposure was associated with reduced ZO-1 expression, whereas *L. plantarum* supplementation partially preserved ZO-1 expression and tight-junction-associated epithelial integrity.

### 3.6. L. plantarum Protects Occludin-Mediated Epithelial Barrier Integrity Under Cadmium Stress

**Figure 6:**
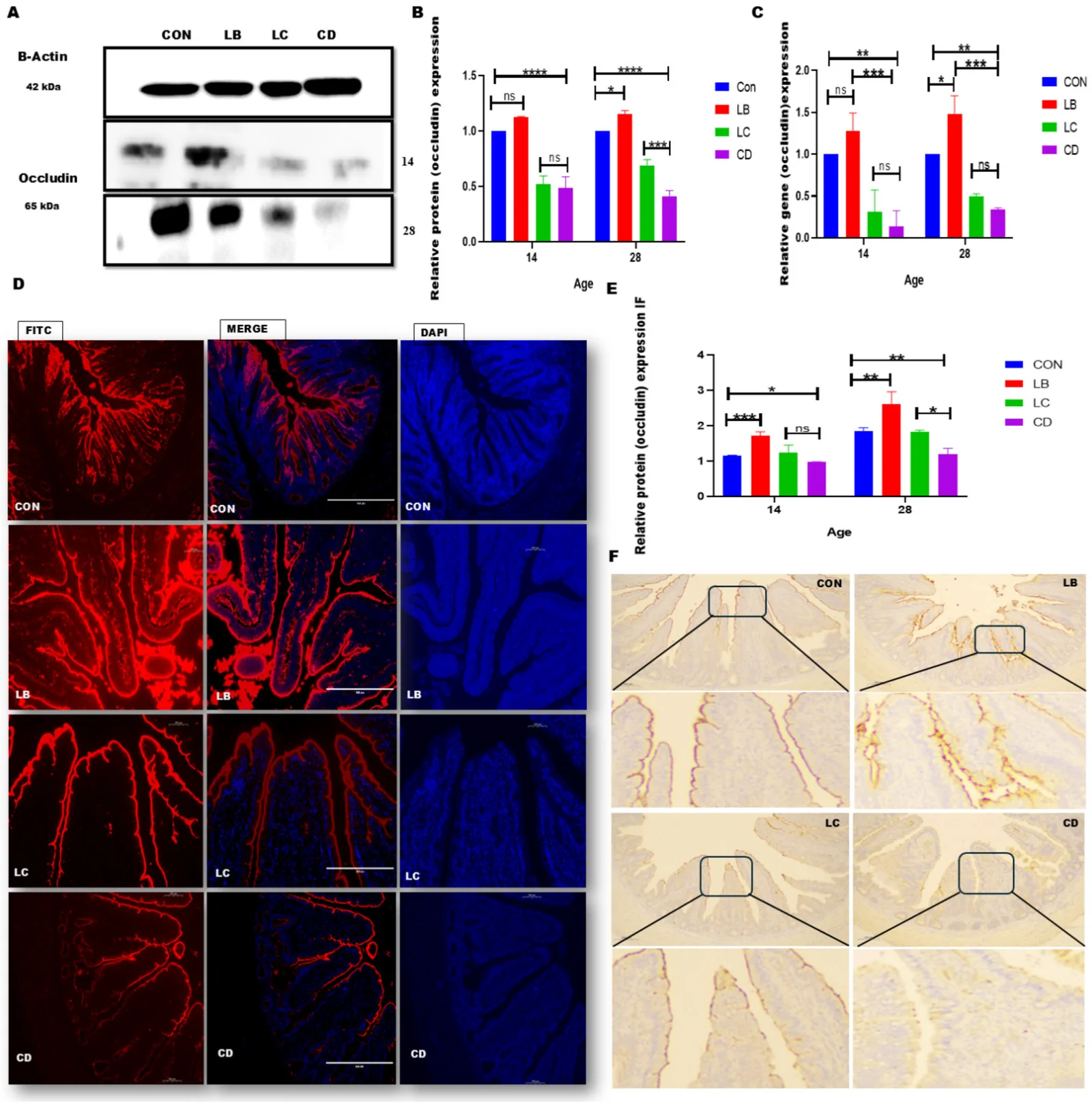
*L. plantarum* maintains occludin expression and localization in the cecum following CdCl_2_. (**A**) Western blot analysis of occludin with β-actin as the loading control (**B**) Densitometric quantification of occludin protein expression (**C**) Relative occludin mRNA expression determined by RT-qPCR (**D**) Representative immunofluorescence images of occludin in cecal sections with DAPI counterstaining; scale bar = 500 µm (**E**) Quantification of occludin immunofluorescence intensity (**F**) Representative Immunohistochemistry staining for occludin; scale bar = 200 µm. Significance level: the values among individual groups are shown as means ± SD * *p* < 0.05, ** *p* < 0.01, *** *p* < 0.001, **** *p* < 0.0001, ns: non-significant.

Occludin, a key transmembrane tight-junction protein, was evaluated to further assess the effects of cadmium exposure on intestinal epithelial barrier integrity. Western blot analysis showed strong occludin expression in the CON and LB groups, whereas cadmium exposure markedly reduced occludin abundance, with the lowest expression observed in the CD group (*p* < 0.001), particularly on day 28. In contrast, the LC group retained higher occludin protein levels than the CD group, indicating partial attenuation of cadmium-associated occludin downregulation by *L. plantarum* supplementation (Figure 6A,B).

A similar trend was observed at the transcriptional level. Occludin mRNA expression was maintained in the CON and LB groups but was significantly suppressed following cadmium exposure. Occludin mRNA expression was significantly lower in the CD group than in the CON (*p* < 0.01). Although occludin mRNA expression showed a numerical increase in the LC group compared with the CD group, this difference did not reach statistical significance, suggesting limited transcriptional recovery. Occludin mRNA expression was significantly higher in the LB group than in the CD (*p* < 0.001), particularly on day 28 (Figure 6C). Immunofluorescence staining further confirmed these findings. The CON and LB groups displayed strong and relatively continuous occludin staining along the epithelial junctions, reflecting preserved barrier organization. In contrast, the CD group exhibited weak and more discontinuous staining, consistent with disruption of junctional organization. The LC group showed an intermediate staining pattern with greater signal continuity than the CD group, indicating the preservation of occludin localization following *L. plantarum* supplementation (Figure 6D,E). Immunohistochemical staining showed a comparable pattern, showing preserved epithelial occludin localization in the CON and LB groups, partial retention in the LC group, and marked loss of staining in the CD group (Figure 6F). Collectively, these findings indicate that cadmium exposure was linked with reduced occludin expression and disrupted epithelial localization, thereby compromising cecal tight-junction integrity. Whereas *L. plantarum* supplementation partially restored occludin expression and junctional organization during cadmium-induced intestinal injury.

### 3.7. L. plantarum Preserves Claudin-1 Expression and Reinforces Cecal Tight-Junction Integrity Under Cadmium Stress

**Figure 7:**
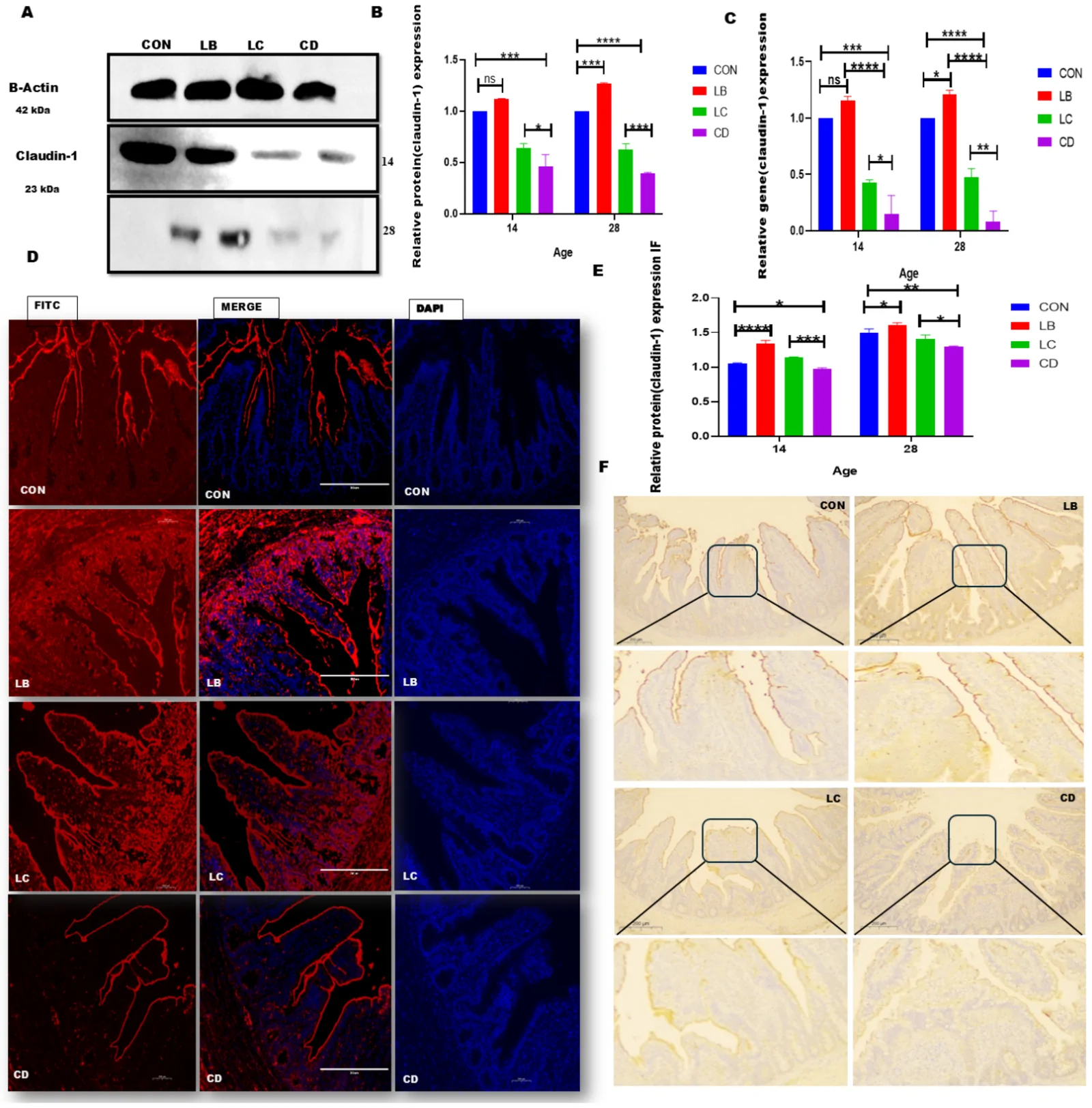
Effects of *L. plantarum* supplementation on claudin-1 expression and preservation of intestinal tight-junction integrity under CdCl_2_ stress. (**A**) Western blot analysis of claudin-1 protein with β-actin as loading control (**B**) Densitometric quantification of claudin-1 protein expression (**C**) Relative claudin-1 mRNA expression determined by RT-qPCR (**D**) Representative Cecal immunofluorescence of claudin-1 with DAPI counterstaining; scale bar = 500 µm (**E**) Quantification of claudin-1 IF intensity (**F**) Representative immunohistochemical staining for claudin-1; scale bar = 200 µm. Significance level: the values among individual groups are shown as means ± SD * *p* < 0.05, ** *p* < 0.01, *** *p* < 0.001, **** *p* < 0.0001, ns: non-significant.

To further assess whether *L. plantarum* protects against cadmium-associated disruption of the intestinal barrier, claudin-1, a key tight-junction protein, was evaluated. Western blot analysis demonstrated that claudin-1 protein expression was maintained in the CON group and was significantly increased in the LB group, specifically on day 28. In contrast, cadmium exposure significantly decreased claudin-1 protein abundance, with the lowest level observed in the CD group. On day 28, claudin-1 protein expression was significantly higher in the LC group compared with the CD group (*p* < 0.001), suggesting that *L. plantarum* could partially restore cadmium-induced claudin-1 loss (Figure 7A,B). A similar trend was observed at the transcriptional level. Claudin-1 mRNA expression was significantly downregulated in cadmium-exposed birds on both day 14 and day 28. On day 28, claudin-1 mRNA expression was significantly higher in the LB group than in the CD group (*p* < 0.0001). In cadmium-exposed birds, *L. plantarum* supplementation markedly increased claudin-1 mRNA expression in the LC group compared with the CD group (*p* < 0.01), particularly on day 28 (Figure 7C). These results were further supported by immunofluorescence staining, which showed relatively strong and continuous claudin-1 staining along the cecal epithelium in the CON and LB groups. In contrast, the CD group exhibited weaker and more discontinuous fluorescence, consistent with altered tight-junction organization. Claudin-1 localization was partially preserved in the LC group as indicated by stronger and more continuous fluorescence than in the CD group (Figure 7D,E). Immunohistochemical staining showed claudin-1 immunoreactivity in the epithelium of the CON and LB groups, partial retention of staining in the LC group, and marked loss of staining in the CD group (Figure 7F). Taken together, these findings demonstrate that cadmium exposure was associated with reduced claudin-1 expression and altered its epithelial localization, consistent with disruption of intestinal barrier integrity. Whereas *L. plantarum* supplementation partially preserved claudin-1 expression and localization. Together with the ZO-1 and occludin findings, these results support a protective effect of *L. plantarum* on multiple components of the cecal epithelial barrier by maintaining the tight-junction complex during cadmium exposure.

## 4. Discussion

Cadmium exposure produced a coordinated intestinal and systemic toxic phenotype in broiler chickens, characterized by impaired growth and survival. It also increased tissue cadmium accumulation, altered tissue calcium homeostasis, and showed variations in hepatic and renal biochemical indices. Also noticed intestinal morphological injury, increased mast-cell infiltration, and altered PIEZO1 and tight-junction protein expression. In contrast, supplementation with *L. plantarum* partially attenuated these alterations, suggesting that preserving intestinal barrier associated with homeostasis contributes to reducing systemic toxicity. These findings are consistent with the well-documented adverse effects of cadmium on the intestinal and systemic physiology of poultry [6,27,31] and with previous evidence that probiotics can alleviate heavy metal toxicity [15,32,33].

A central finding of the present study was a reduction in tissue cadmium accumulation following *L. plantarum* supplementation, which was accompanied by improved growth, survival, and hepatic and renal biochemical parameters. These systemic improvements coincided with the preservation of cecal morphology, reduced mast-cell abundance, and maintenance of tight-junction proteins ZO-1, occludin, and claudin-1. These findings suggest that the reduced tissue cadmium burden and preservation of intestinal barrier-associated integrity may have contributed to the attenuation of systemic effects of cadmium exposure. Additionally, *L. plantarum* supplementation significantly normalized tissue calcium homeostasis and reduced PIEZO1 upregulation. Instead, they propose that there is a correlation between calcium dysregulation, increased expression of PIEZO1, and intestinal barrier damage [25,34,35]. Thus, the concurrent changes in calcium homeostasis, PIEZO1 expression, and intestinal barrier-associated proteins support an association-based working model rather than a direct causal relationship because PIEZO1 activity was not directly manipulated.

Systemic biochemical findings further support an association between reduced cadmium burden and attenuation of toxicity. Cadmium exposure was associated with increased serum ALT, AST, ALP, and creatinine concentrations and altered total protein levels, consistent with the recognized hepatotoxic and nephrotoxic effects of cadmium [36,37,38,39,40]. *L. plantarum* supplementation partially attenuated these biochemical alterations and reduced tissue cadmium accumulation, suggesting that a lower cadmium burden was associated with reduced systemic toxicity. This finding is compatible with previous studies reported ability of *Lactobacillus* species that they can interact with heavy metals through cell-wall components to bind cadmium, potentially reducing their intestinal availability [41]. These concurrent changes suggest that lowering the cadmium burden may contribute to the observed improvement in systemic biochemical status. Therefore, the lower tissue cadmium concentration may suggest lowered intestinal availability, enhanced elimination, altered tissue distribution, or other host- or bacteria-mediated processes. The correlation analyses further showed that several organ indices and biochemical parameters changed in parallel; however, because these variables were simultaneously influenced by the experimental treatments, the correlations should be interpreted as descriptive associations rather than evidence of causality.

Maintenance of intestinal barrier-associated integrity may also have contributed to the observed systemic protection. Previous studies have linked disruption of intestinal epithelial integrity with increased permeability and enhanced translocation of luminal toxicants and inflammatory mediators, which may contribute to systemic and organ injury [10,27]. In the present study, *L. plantarum* supplementation was associated with preservation of cecal morphology, reduced mast cell abundance, and maintenance of ZO-1, occludin, and claudin-1 expression and localization. These findings suggest that preservation of intestinal homeostasis may be an important component of the protective effects of *L. plantarum* during cadmium exposure.

A key finding of the present study was the concurrent alteration of serum and cecal calcium concentrations and increased intestinal PIEZO1 expression following cadmium exposure. Cadmium is recognized as a calcium-mimicking toxic metal that can interfere with calcium transport and intracellular calcium regulation, resulting in abnormal calcium influx, disturbed cellular signaling, and mitochondrial dysfunction [42,43,44]. Cadmium-induced intracellular calcium imbalance appears to promote abnormal activation of the mechanosensitive ion channel PIEZO1, thereby disturbing epithelial mechano-transduction and compromising the structural organization of the intestinal barrier [24,42,45]. Consistent with these reported effects, cadmium exposure in the present study was associated with altered calcium concentrations in serum and cecal tissue, together with increased PIEZO1 expression at both the transcriptional and protein levels. PIEZO1 functions as a mechanosensitive, calcium-permeable ion channel involved in epithelial mechano-transduction, intracellular calcium regulation, and barrier homeostasis; excessive calcium accumulation may contribute to its upregulation under cadmium-induced cellular stress [45,46]. Sustained upregulation of PIEZO1-associated calcium signaling may induce cytoskeletal remodeling and destabilization of the tight-junction complex, coinciding with reduced expression and altered localization of ZO-1, occludin and claudin-1, ultimately weakening epithelial barrier function [24,25,47]. Whereas *L. plantarum* partially normalized these alterations in calcium status and PIEZO1 expression. These concurrent changes suggest a possible relationship between altered calcium homeostasis, PIEZO1 expression, and intestinal barrier-associated alterations. Therefore, the relationship between calcium homeostasis, PIEZO1 expression, and intestinal barrier integrity should be interpreted as an association rather than a demonstrated causal pathway.

Cadmium exposure significantly affected cecal morphology, resulting in decreased villus height, increased crypt depth, and a decreased villus height-to-crypt depth ratio. These changes are consistent with mucosal injury and compensatory remodeling of the epithelium [10,21]. In contrast, these morphometric parameters were remarkably reversed through *L. plantarum* supplementation, indicating preservation of intestinal architecture. Cadmium exposure also significantly increased mast cell infiltration into the cecal mucosa, suggesting an altered local inflammatory response. The histamine, proteases and cytokines released by the activated mast cells can exacerbate epithelial damage and increase intestinal permeability [48,49]. The lower mast-cell abundance observed after *L. plantarum* supplementation was therefore associated with reduced mucosal inflammation; however, mast-cell mediators were not directly measured in this study.

These morphological and inflammatory changes were accompanied by reduced expression and altered localization of ZO-1, occludin and claudin-1. These proteins are important components of epithelial tight-junction complexes and contribute to the regulation of paracellular permeability and epithelial barrier organization [10,47]. In the present study, their mRNA and protein expression were downregulated following cadmium exposure, whereas *L. plantarum* supplementation preserved their expression and localization [21,47]. The parallel improvement in intestinal morphology, mast cell infiltration and tight junction-associated proteins supports an association between *L. plantarum* supplementation and preservation of epithelial junctional integrity. In addition to improving intestinal barrier function, *L. plantarum* also maintained the coordinated organization of tight junction structures, which may contribute to reduced intestinal permeability, diminished inflammatory responses, and improved systemic physiological homeostasis.

Collectively, the findings of the present study support an association-based working model in which cadmium exposure is accompanied by altered calcium homeostasis, increased PIEZO1 expression, intestinal morphological injury, and disruption of tight-junction-associated proteins. *L. plantarum* supplementation partially normalized these concurrent alterations and was associated with reduced tissue cadmium accumulation and improved intestinal and systemic outcomes.

Conversely, dietary supplementation with *L. plantarum* was associated with improved calcium homeostasis, downregulation of PIEZO1, preservation of tight-junction organization, and reduced mast cell accumulation. Previous studies have shown that *lactobacilli* species can bind heavy metals via negatively charged cell wall components such as peptidoglycan, teichoic acids and surface polysaccharides, thereby potentially decreasing intestinal cadmium uptake and limiting its systemic bioavailability [33,50]. Our findings are consistent with a potential contribution of this process to the lower tissue cadmium concentration observed following *L. plantarum* supplementation. At the same time, it preserves the organization of tight junctions and decreases mucosal inflammation, thereby contributing to epithelial barrier function. Maintenance of intestinal barrier integrity is now recognized as a key factor in gut-liver communication, and disruption of the epithelial barrier has been linked to systemic inflammation and secondary injury to the liver and kidneys [10]. Thus, liver and renal function are likely related to better intestinal homeostasis rather than just to direct protection of these organs.

Overall, these results suggest that *L. plantarum* can alleviate several adverse effects of cadmium by regulating intestinal barrier function. The intestine may represent a key therapeutic target for probiotic intervention during heavy metal exposure. The findings of the current study provide valuable insights into the potential mechanisms underlying cadmium-induced intestinal barrier dysfunction and suggest that *L. plantarum* may serve as an effective nutritional strategy to mitigate the harmful effects of heavy metal exposure in poultry. Our results indicate that concurrent alteration in calcium homeostasis and PIEZO1 expression and associated tight junction-associated proteins, together with observed physiological, biochemical, molecular, histological and immunological evidence, support an association between these factors and intestinal barrier disruption during cadmium exposure. Maintenance of the tight-junction complex and preservation of epithelial integrity may therefore represent key therapeutic targets for preventing not only intestinal injury but also subsequent systemic complications associated with environmental heavy metal contamination. The present study has several limitations that should be considered when interpreting the findings. First, only one dietary CdCl_2_ concentration and one dose of *Lactobacillus plantarum* were evaluated. Therefore, dose-dependent relationships between cadmium exposure, PIEZO1 expression, and intestinal-barrier injury could not be established. The minimum effective and optimal protective doses of *L. plantarum* also remain to be determined. In addition, the dietary concentration of 80 mg/kg CdCl_2_ was selected to induce reproducible toxicity within the 28-day experimental period and represents a controlled high-dose exposure model. Consequently, the magnitude of the observed injury and protective effects should not be directly extrapolated to lower, prolonged, or variable cadmium exposures under commercial poultry-production conditions.

The concurrent changes in tissue calcium concentration, PIEZO1 expression, intestinal morphology, and tight-junction protein levels indicate biologically relevant associations but do not establish a direct causal relationship. PIEZO1 channel activity and intracellular calcium flux were not functionally evaluated. Therefore, these findings should not be interpreted as direct evidence of intracellular calcium influx or PIEZO1 activation.

The current manuscript was designed to focus on growth performance, systemic biochemical alterations, intestinal morphology, mast-cell responses, calcium status, PIEZO1 expression, and tight-junction integrity. Complementary analyses, including endoplasmic-reticulum-stress responses, 16S rRNA-based intestinal microbial profiling, and liver histopathology, were conducted as part of the second phase of the broader investigation and are being evaluated separately. Accordingly, the present manuscript does not provide an integrated assessment of microbiota-related responses, endoplasmic-reticulum stress, or hepatic injury.

Future studies should evaluate multiple cadmium concentrations, different *L. plantarum* doses, longer exposure periods, and environmentally relevant models. Functional validation using PIEZO1-specific pharmacological inhibitors, gene silencing or genetic modification, intracellular calcium imaging, electrophysiological assessment, and intestinal-permeability measurements would help determine whether PIEZO1 directly contributes to cadmium-induced intestinal-barrier dysfunction or *L. plantarum*-associated protection. Integrating the present findings with 16S rRNA sequencing, endoplasmic-reticulum-stress markers, liver histopathology, metabolomic analysis, cadmium-binding assays, and fecal cadmium-excretion measurements may further clarify the mechanisms underlying the protective effects of *L. plantarum*.

## 5. Conclusions

In conclusion, the use of *L. plantarum* reduced cadmium-induced intestinal-barrier damage and systemic toxicity in broiler chickens. The protective response led to decreased accumulation of cadmium in tissues and maintenance of intestinal homeostasis and physiological performance. The concurrent normalization of calcium homeostasis and PIEZO1 expression suggest a possible association between calcium-sensitive signaling and intestinal barrier protection; however, direct involvement of PIEZO1 requires functional confirmation. These findings support further investigation of *L. plantarum* as a dietary strategy for reducing cadmium toxicity in poultry.

Collectively, these findings support an association-based model in which cadmium exposure is accompanied by disturbed calcium homeostasis, increased PIEZO1 expression, and impaired intestinal-barrier integrity. *L. plantarum* supplementation partially normalized these concurrent alterations. However, because PIEZO1 activity was not directly measured, inhibited, or genetically manipulated, the present study cannot establish whether PIEZO1 mediates cadmium-induced barrier injury or the protective effects of *L. plantarum*. Functional studies are required to test this proposed relationship.

These results confirmed the preliminary experimental evidence that the strain *L. plantarum* might be beneficial in minimizing intestinal and systemic damage in broilers induced by cadmium. However, more research under multiple doses, more birds, longer duration of exposure, and under field conditions is needed before application in poultry production is advisable.

## Figures and Tables

**Table 1:** Primer sequences.

Primer Name	Forward	Reverse
ZO-1	5′-CGCAGTCGTTCACGATCT-3′	5′-GGAGAATGTCTGGAATGGTCTG-3′
Occludin	5′-GCTCCCGGCTGCCATT-3′	5′-CGTCGTCCACGTAGTAGGAG-3′
Claudin1	5′-TGGCCACGTCATGGTATGG-3′	5′-AACGGGTGTGAAAGGGTCATAG-3′
PIEZO1	5′-GAGAGCTGCAGAATGGCTCC -3′	5′-GGTCGGTGGTGTGCTTGTC-3′
GAPDH	5′-AGAACATCATCCCAGCGT-3′	5′-AGCCTTCACTACCCTCTTG-3′

**Table 2:** Primary antibody sequences.

Antibody Name	Protein Name	Catalog Number	Supplier
Anti-β-actin	β-actin	AC026	ABclonal
FAM38A/PIEZO1	PIEZO1	A23380	ABclonal
Anti-Claudin	Claudin-1	WL03073	Wen li Bio
Anti-Occludin	Occludin	WL01996	Wen li Bio
Anti-ZO-1	ZO-1	WL03419	Wen li Bio

## Data Availability

The raw data supporting the conclusions of this article will be made available by the authors on request.
